# Selective deletion of connective tissue growth factor attenuates experimentally-induced pulmonary fibrosis and pulmonary arterial hypertension

**DOI:** 10.1016/j.biocel.2021.105961

**Published:** 2021-05

**Authors:** Angela Y.Y. Tam, Amy L. Horwell, Sarah L. Trinder, Korsa Khan, Shiwen Xu, Voon Ong, Christopher P. Denton, Jill T. Norman, Alan M. Holmes, George Bou-Gharios, David J. Abraham

**Affiliations:** a Centre for Rheumatology and Connective Tissue Disease, Department of Inflammation, Division of Medicine, University College London, London, NW3 2PF, UK; b Department of Musculoskeletal and Ageing Science, Institute of Life Course and Medical Sciences, University of Liverpool, Liverpool, L7 8TX, UK; c Department of Renal Medicine, Division of Medicine, University College London, London, NW3 2PF, UK

**Keywords:** Floxed, Flanked by loxP sites, Connective tissue grown factor (CTGF, CCN2), Interstitial lung disease (ILD), Pulmonary fibrosis, Pulmonary arterial hypertension (PAH), Bleomycin-induced fibrosis, Systemic sclerosis (scleroderma, SSc)

## Abstract

Connective tissue growth factor (CTGF, CCN2) is a matricellular protein which plays key roles in normal mammalian development and in tissue homeostasis and repair. In pathological conditions, dysregulated CCN2 has been associated with cancer, cardiovascular disease, and tissue fibrosis. In this study, genetic manipulation of the CCN2 gene was employed to investigate the role of CCN2 expression *in vitro* and in experimentally-induced models of pulmonary fibrosis and pulmonary arterial hypertension (PAH). Knocking down CCN2 using siRNA reduced expression of pro-fibrotic markers (fibronectin p < 0.01, collagen type I p < 0.05, α-SMA p < 0.0001, TIMP-1 p < 0.05 and IL-6 p < 0.05) in TGF-β-treated lung fibroblasts derived from systemic sclerosis patients. *In vivo* studies were performed in mice using a conditional gene deletion strategy targeting CCN2 in a fibroblast-specific and time-dependent manner in two models of lung disease. CCN2 deletion significantly reduced pulmonary interstitial scarring and fibrosis following bleomycin-instillation, as assessed by fibrotic scores (wildtype bleomycin 3.733 ± 0.2667 vs CCN2 knockout (KO) bleomycin 4.917 ± 0.3436, p < 0.05) and micro-CT. In the well-established chronic hypoxia/Sugen model of pulmonary hypertension, CCN2 gene deletion resulted in a significant decrease in pulmonary vessel remodelling, less right ventricular hypertrophy and a reduction in the haemodynamic measurements characteristic of PAH (RVSP and RV/LV + S were significantly reduced (p < 0.05) in CCN2 KO compared to WT mice in hypoxic/SU5416 conditions). These results support a prominent role for CCN2 in pulmonary fibrosis and in vessel remodelling associated with PAH. Therefore, therapeutics aimed at blocking CCN2 function are likely to benefit several forms of severe lung disease.

## Highlights

• CCN2 is selectively and exquisitely induced by the TGF-β signalling pathway.
• CCN2 appears to mediate many of the activities characteristic of TGF-β.
• Inhibition of CCN2 in vitro reduces many of the functional pro-fibrotic activities of TGF-β.
• Fibroblast-specific deletion of CCN2 attenuates interstitial fibrosis and pulmonary vascular remodelling in models of lung diseases.

## A1 Introduction

Interstitial lung disease (ILD) encompasses a group of disorders involving inflammation and fibrosis of lung tissue, which can be idiopathic (IPF), or develop in the context of connective tissue diseases such as systemic sclerosis (SSc) (Chambers and Mercer 2015; Denton CP, Khanna 2017). There is significant morbidity and mortality associated with IPF, an ILD with a median survival of only 3–5 years post-diagnosis (Gibson et al. 2020). Similarly, pulmonary involvement, which includes pulmonary hypertension (PH) and pulmonary fibrosis, is the leading cause of death in patients with SSc, with extent of ILD being positively associated with mortality (Nihtyanova and Denton, 2020). Therefore, despite some advances in treatments (Denton and Khanna, 2017; Wongkarnjana et al., 2020), there is still an unmet need to develop more effective therapies for fibrotic disease, which requires elucidation of the pathobiology and molecular mechanisms underlying pulmonary fibrosis and pulmonary vascular disease.

A common feature amongst the severe pulmonary diseases, and indeed other remodelling diseases affecting other target organs including the heart, liver and kidney, is the presence of inflammation as a key driver or modifier of disease, promoting pro-fibrotic events leading to tissue remodelling, scarring and replacement fibrosis. Major pathways that have been highlighted to be involved in these latter events are the TGF-β and CCN2 pathways. These pathways have been studied at the molecular level, and in a variety of disease settings including renal fibrosis, lung disease and skin fibrosis (Henrot et al., 2019; Leask, 2017; Miller et al., 2019; Qi et al., 2008; Wang et al., 2019; Yin and Liu, 2019). In particular, aberrant expression of connective tissue growth factor (CTGF, CCN2) has been extensively explored (Abraham, 2008; Adler et al., 2010; Ramazani et al., 2018; Szabo et al., 2014).

Multiple signalling pathways and cytokines, including transforming growth factor-β (TGF-β), can induce CCN2 expression in various cell types (Takigawa, 2018). Fibroblasts are a key effector cell type in fibrosis and a major source of CCN2, although in adults, CCN2 expression is normally tightly regulated and limited to the process of wound healing. However, in fibrotic lesions, CCN2 is often constitutively overexpressed (Abraham, 2008; Leask, 2017). With respect to SSc, increased CCN2 expression has been detected in patient groups, notably in blister fluids, sera, bronchoalveolar lavage fluids, skin and lung tissues, where CCN2 levels have been shown to correlate with clinical parameters, making CCN2 useful as a biomarker (Dziadzio et al., 2005; Hasegawa, 2016; Igarashi et al., 1995; Sato et al., 2000; Shi-wen et al., 2000).

As a member of the CCN family of matricellular proteins, CCN2 can interact with specific receptors (including integrins, heparan sulphate proteoglycans, low-density lipoprotein receptor-related proteins and neurotrophic tyrosine kinase receptor type 1), ECM proteins (including fibronectin and perlecan) and cytokines (including bone morphogenetic proteins, TGF-β and vascular endothelial growth factor), in order to modulate cellular functions and signalling (Jun and Lau, 2011). The exact mechanisms underlying the pro-fibrotic actions of CCN2 are not yet fully understood, although CCN2 is known to act as a co-factor in enhancing the fibrotic activity of TGF-β, and can promote ECM deposition and remodelling (Leask and Abraham, 2006). Previous studies have shown that fibroblast-specific overexpression of CCN2 *in vivo* results in a fibrotic phenotype, characterised by increased collagen deposition in the skin, lung and kidney (Doherty et al., 2010; Ponticos et al., 2009; Sonnylal et al., 2010). CCN2 also contributes to the expansion of the pool of pro-fibrotic cells by inducing the differentiation of tissue-resident fibroblasts, and transdifferentiation of other cell types such as epithelial cells and fibrocytes, into activated fibroblasts, or myofibroblasts (Pakshir et al., 2020; Sonnylal et al., 2013). This may explain the association of CCN2 expression with expression of α-smooth muscle actin (SMA), a marker of myofibroblasts.

A substantial amount of the literature available on pulmonary fibrosis is derived from studies *in vitro* or in animal models. This reflects the challenges of obtaining human lung tissue for research, as the procedure of open lung biopsies is becoming increasingly rare, especially in the early stages of disease. One of the most common *in vivo* models of pulmonary fibrosis uses bleomycin to induce acute lung injury in mice. Bleomycin induces cleavage of DNA strands, resulting in pulmonary inflammation and interstitial fibrosis (Walters and Kleeberger, 2008). In this and other animal models of fibrosis, CCN2 and TGF-β work together to produce a fibrotic response (Wang et al., 2011). The fibroblasts present in bleomycin‐induced fibrotic lesions express phosphorylated Smad3, indicative of TGF-β signalling (Takagawa et al., 2003), and increased CCN2 expression (Howell et al., 2001; Liu et al., 2010), which further supports the importance of CCN2 as a pro-fibrotic mediator. Therefore, impairing the availability or activity of CCN2 represents a potential strategy for inhibiting or reversing tissue remodelling and fibrosis. The objective of this study is to investigate the effects of CCN2 gene silencing and CCN2 deletion on the development of a disease phenotype using fibroblast cultures and two experimentally-induced models of lung disease: bleomycin-induced pulmonary fibrosis and the chronic hypoxia/Sugen model of pulmonary arterial hypertension (PAH). In view of the critical roles of CCN2 in growth and development, and the observation that conventional homozygous CCN2-null mice are neonatal lethal, due to impaired chondrogenesis and ECM abnormalities (Ivkovic et al., 2003), we employed conditionally-inducible CCN2-null mice (Liu et al., 2011) within these studies.

## 2 Materials and methods

### 2.1 Cell culture

Human lung fibroblast cultures were established from lung biopsies of patients with diffuse cutaneous SSc, and healthy volunteers, as described previously (Abraham et al., 1991). All patients fulfilled the American College of Rheumatology / European League against Rheumatism criteria for the diagnosis of SSc (van den Hoogen et al., 2013). Informed consent and ethical approval were obtained for all procedures (Ethical Approval: Health Research Authority, NRES Committee London- Hampstead, Research Ethics Committee (REC) reference: 6398). Fibroblasts were cultured in Dulbecco’s Modified Eagle Medium supplemented with fetal calf serum (10 %), penicillin (100 units/mL) and streptomycin (100 mg/mL; Life Technologies Inc.) and incubated at 37 °C in a humidified atmosphere of 5% CO_2_ in air. All cells were used between passages 2–6 for experiments. Confluent monolayers were made quiescent by incubation in 0.5 % bovine serum albumin (BSA)–supplemented DMEM for 24 h before lysis for Western blotting

### 2.2 CCN2 siRNA knockdown

Human lung fibroblasts were cultured to 60–70% confluence in antibiotic-free complete medium. Cells were incubated for 72 h in the presence of non-targeting control siRNA sequences (siNTC; 50 nM; Dharmacon) or a pool of various siRNA sequences targeting CCN2 mRNA (siCCN2; 50 nM; Dharmacon), in Dharmafect transfection reagent (Dharmacon). CCN2 target sequences used were: ACAAUGACAUCUUUGAAUC, AGGAAGAUGUACGGAGACA, CGAUUAGACUGGACAGCUU, GAGAGACAUUAACUCAUUA. Cells were incubated for a further 24 h with the addition of TGF-β (4 ng/mL) before being lysed for Western blot analysis.

### 2.3 Western blot analysis

Fibroblasts were lysed in radioimmunoprecipitation assay buffer (RIPA) buffer (Sigma) with protease inhibitors (Roche). Total protein concentration was determined by BCA assay (Pierce). Samples were heat-denatured under reducing conditions with NuPAGE reducing agent (Invitrogen) and subjected to the NuPAGE electrophoresis system (Invitrogen). Proteins were transferred onto nitrocellulose membranes (GE Healthcare), which were incubated in block buffer (5% non-fat dry milk, 0.1 % Tween-20 (Sigma) in phosphate-buffered saline) for 1 h, followed by incubation at 4 °C overnight with primary antibodies against α-SMA (71 ng/mL, Dako), collagen type I (0.4 μg/mL, Millipore), CCN2 (0.1 μg/mL, Santa Cruz Biotechnology), EDA-fibronectin (2 μg/mL, Sigma-Aldrich), tissue inhibitor of metalloproteinase 1 (TIMP-1; 0.1 μg/mL, Abcam), and β-tubulin (0.2 μg/mL, Abcam), diluted in block buffer (5% non-fat dry milk, 0.1 % Tween-20 (Sigma) in phosphate-buffered saline). Blots were developed by incubation with horseradish peroxidase (HRP)-conjugated secondary antibodies (1:5000, Cell Signalling). The signal was detected using enhanced chemiluminescence (ECL; Pierce) and exposure to Hyperfilm (GE Healthcare). Densitometry was performed on the bands using Visionwork LS software, and displayed in arbitrary densitometry units (ADU).

### 2.4 Enzyme-linked immunosorbent assay (ELISA) IL-6 in fibroblast conditioned media

Conditioned media were aspirated from fibroblast monolayers and centrifuged (350 x g, 5 min) to remove dead cells and debris. Human IL-6 concentration in the media was measured using the DuoSet ELISA Development kit, as recommended by the manufacturer (R&D Systems). Values were normalised to total protein concentration for each cell culture, as determined by BCA assay.

### 2.5 Double transgenic CCN2-floxed with Col1α2-CreER inducible CCN2 knockout mice

All mice used for experiments were between 6–12 weeks of age at the beginning of the study, including males and females. Animals were genotyped by PCR. Mice harbouring a floxed CCN2 allele have been described previously (Liu et al., 2010). Homozygous CCN2-floxed mice were crossed with mice positive for Col1α2-CreER or Col1α2-CreER^T2^ (Denton et al., 2003), which express a tamoxifen-inducible Cre recombinase driven by the 2.3-kb mouse collagen type I, alpha 2 (Col1α2) promoter. CCN2 gene deletion was achieved via cre-recombinase expression, induced by intraperitoneal injection with tamoxifen (1 mg/50 μL saline; Sigma) (Liu et al., 2011).

### 2.6 Col1α-R26TmG fibroblast-specific Cre reporter mice

Generation of a fibroblast-specific Cre-inducible strain was described previously (Li et al., 2017). Col1α-R26TmG mice harbour the fibroblast-specific enhancer, Col1α-CreER^T2^ and a double fluorescent Cre reporter construct (Muzumdar et al., 2007). This construct expresses membrane-targeted tandem dimer Tomato (tdTomato; red signal) before Cre-mediated excision, and enhanced green fluorescent protein (EGFP; green signal) after excision, enabling the assessment of cell morphology and tamoxifen-inducible Col1α-CreER^T2^ activity.

### 2.7 Bleomycin murine model of pulmonary fibrosis

The bleomycin-induced murine model was performed by administration of bleomycin (0.12 units (U) per mouse) or saline via oropharyngeal instillation, as described previously (Ponticos et al., 2009). After 14–21 days (treatment time as indicated in each figure), lung tissue was harvested for histology or fixed by inflation for micro-CT imaging.

### 2.8 Hypoxia/SU5416 murine model of pulmonary arterial hypertension (PAH)

Mice were treated as previously described (Ciuclan et al., 2011; Derrett-Smith et al., 2013; Good et al., 2015). After 21 days, mice were anesthetized to allow measurement of right ventricular systolic pressure (RVSP) and mean arterial aortic pressure using a catheter and PowerLab system (AdInstruments), and lung tissue taken for histological analysis as for the bleomycin model.

### 2.9 Micro-computed tomography (micro-CT) imaging of mouse lungs *ex vivo*

Lungs were manually inflated prior to removal before being suspended in 3 % potassium Iodide (KI) and fixed in 10 % neutral buffered formalin (NBF) overnight at 4 °C. Lungs were then dehydrated through a series of graded ethanols: 70 %, 80 %, 90 %, 100 %, 100 %, for 1.5 h in each solution, suspended in hexamethyldisilazane (HMDS) (Sigma Aldrich) for 1.5 h and dried in a fume hood for 2 h. A Bruker Skyscan 1272 ex vivo micro-CT system was used to scan lungs at a resolution of 20 μm, using a 0.25 mm aluminium filter and 0.3° rotation step. Images were reconstructed using Skyscan Nrecon software and visualised using DataViewer.

### 2.10 Reverse transcription quantitative PCR (RT-qPCR)

RNA was extracted from whole lungs, after micro-CT scanning. qPCR was performed using the SensiMIX™ SYBR® Hi-ROX kit (Bioline) and analysed on a Corbett Rotor Gene RG-6000 using cycling parameters: initial hold for 10 min at 95 °C, 40 cycles of 95 °C for 10 s, 60 °C for 15 s, 72 °C for 20 s, and acquisition on the green channel. Rotor-Gene 6000 series software version 1.7 was used to analyse the results. ΔΔCt values were calculated and normalised to the 18S rRNA reference gene.

### 2.11 Histology and modified Ashcroft Scale

Mouse lung tissue was fixed in formalin and embedded in paraffin. Tissue blocks were sectioned (4−6 μm) and mounted on glass slides for haematoxylin and eosin (H&E; Surgipath and Sigma) and PicroSirius Red staining (VWR and Raymond Lab) or Goldner trichrome, for general morphological analysis and collagen deposition, respectively (Thoua et al., 2012). Sections were imaged using the NanoZoomer (Hamamatsu), and analysed using 10X magnification in the NDP View software, or imaged using a Zeiss Axio Observer apotome microscope and Axio Cam MR R3 camera and viewed in ZEN Blue microscopy software. The modified Ashcroft score was used to quantify the extent of fibrosis, as described previously (Hubner et al., 2008). Multiple fields were scored following a raster-like pattern until the entire section was covered. The sum of the grades was divided by the number of fields to obtain a fibrotic index. In every field, the predominant degree of fibrosis was recorded as that occupying more than half of the field area while areas dominated by bronchial or tracheal tissue were omitted.

### 2.12 Immunohistochemistry and quantitation of DAB staining

Paraffin-embedded mouse lung tissues were prepared as described above. Formalin fixed 3 μm paraffin sections were used for immunostaining. Heat-mediated antigen retrieval was performed in citrate buffer (10 mM citric acid, pH 6.0). Endogenous peroxidase was blocked using 0.5 % hydrogen peroxide. Normal horse serum (2.5 %) or normal goat serum (2.5 %) was used to block tissues for 1 h, before incubation with anti-CCN2 antibody (4 μg/mL, rabbit polyclonal, Abcam) or anti-fibronectin antibody (250 ng/mL, mouse IgG, BD Transduction), α-SMA (100 ng/mL, Dako, mouse IgG), diluted in antibody diluent (Dako). Sections were the incubated with anti-rabbit ImmPress Polymer reagent (Vector Laboratories) or goat anti-mouse HRP (Dako), respectively, before incubating with DAB peroxidase substrate solution and counterstained with Meyer’s haematoxylin (Sigma). Specificity of staining was confirmed by incubation sections with an isotype-matched rabbit IgG (Dako) or mouse IgG1 (Santa Cruz) control antibody. Sections were imaged using the NanoZoomer (Hamamatsu), and four images at 20X magnification were captured for each tissue section in the NDP View software and exported as tif-image files. Staining was semi-quantitatively assessed by measuring the DAB stain intensity and normalising against the number of haematoxylin-stained nuclei for each image in Fiji (Crowe and Yue, 2019; Schindelin et al., 2012).

### 2.13 Statistical analysis

Statistical significance to compare groups was calculated by one-way ANOVA or unpaired student two-tailed *t*-test, as indicated, using Microsoft Excel or GraphPad Prism V8.43. A value of p < 0.05 was considered significant.

## 3 Results

### 3.1 CCN2 siRNA knockdown attenuated expression of fibrotic protein markers in SSc lung fibroblasts

**Fig. 1:**
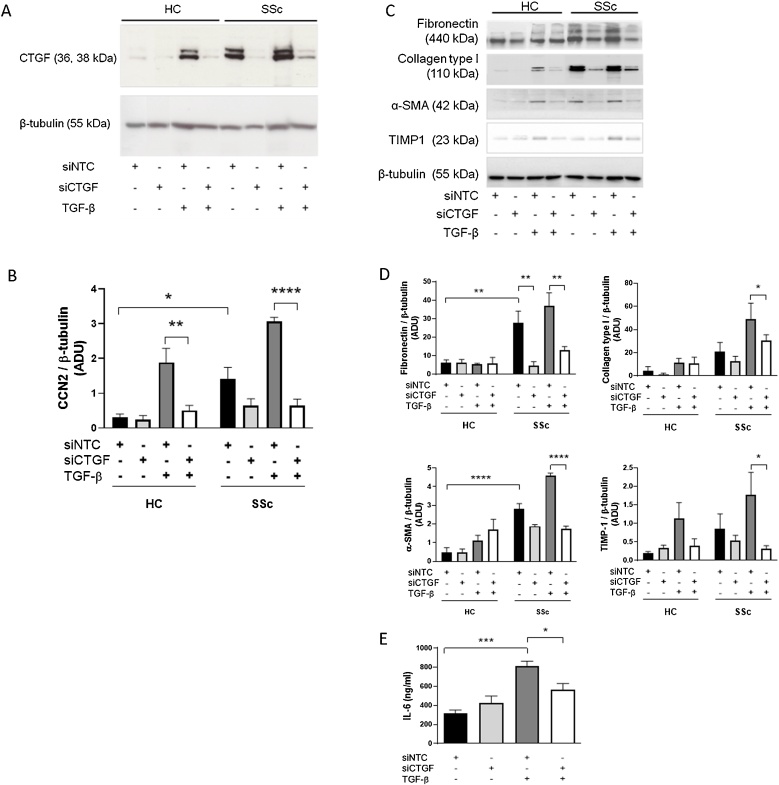
CCN2 siRNA attenuates expression of fibrotic protein markers in SSc lung fibroblasts in vitro. (A&C) Lung fibroblasts isolated from healthy controls (HC; n = 3) and scleroderma (SSc; n = 3) patients were cultured with recombinant TGF-β (4 ng/mL) to stimulate CCN2 expression, and with CCN2-specific siRNA (siCCN2) to knockdown CCN2 expression. Non-target control siRNA (siNTC) was used as negative control. Expression of CCN2 and other fibrotic proteins including fibronectin, collagen type I, α-smooth muscle actin (SMA) and tissue inhibitor of metalloproteinase 1 (TIMP1), were analysed by Western blotting, and normalised to expression of β-tubulin. (B&D) Densitometry analysis of Western blots. (E) Conditioned media collected from TGF-β-treated HC fibroblast cultures were analysed by enzyme-linked immunosorbent assay (ELISA), for quantitation of secreted IL-6, normalised to total protein in the cell layers, as determined by BCA assay. Bars show mean ± SEM. Statistical significance was tested by one-way ANOVA with Sidak multiple comparison, * p < 0.05, ** p < 0.01, *** p < 0.001.

In order to investigate CCN2 expression in human lung fibroblasts, cell lysates of cultured primary fibroblasts derived from lung tissue of HC individuals and SSc patients (n = 3 biological replicates per group) were analysed by Western blotting and one-way ANOVA with Sidak post hoc test. CCN2 expression was statistically significantly higher in SSc lung fibroblasts (1.42 ± 0.561 ADU, p = 0.0108; Fig. 1A) than HC fibroblasts (0.606 ± 0.166 ADU). Furthermore, to determine the role of CTGF as a fibrotic mediator, siRNA treatment was used to examine the effects of knocking down CCN2 on the expression of fibrotic markers in lung fibroblasts. Transfection of HC lung fibroblasts with siCCN2 significantly decreased TGF-β-induced CCN2 expression (0.508 ± 0.253 ADU, p = 0.0019) compared to transfection with siNTC (1.89 ± 0.700 ADU). Expression of CCN2 in HC fibroblasts not treated with TGF-β were unchanged with siCTGF treatment as basal levels were low. In SSc lung fibroblasts, there was a trend towards decreased basal CCN2 expression with siCCN2 treatment compared to siNTC treatment, although this was not statistically significant (p = 0.097). This may be due to the variability in basal CCN2 expression in different SSc cell lines. Despite this, a decrease in basal fibronectin expression was observed with siCCN2 treatment compared to siNTC treatment, even in SSc fibroblasts not treated with TGF-β (4.62 ± 3.77 vs 27.9 ± 10.8 ADU, p = 0.002). Additionally, knockdown with siCCN2 did significantly lower TGF-β-induced CCN2 expression (0.651 ± 0.316 ADU, p < 0.0001; Fig. 1A&B). Concomitantly, TGF-β-induced expression of several profibrotic markers in these SSc fibroblasts, including fibronectin (12.8 ± 3.77 vs 37.0 ± 12.2 ADU, p = 0.0014), collagen type I (27.4 ± 4.37 vs 51.7 ± 21.6 ADU, p = 0.0469), TIMP-1 (0.314 ± 0.148 vs 1.77 ± 1.06 ADU, p = 0.0192) and α-SMA (1.76 ± 0.230 vs 4.89 ± 0.245 ADU, p < 0.0001; Fig. 1C&D), were statistically lower with siCCN2-treatment than with siNTC treatment. Similarly, secreted IL-6 expression was lower in siCCN2-treated compared to siNTC-treated HC lung fibroblasts (564 ± 112 vs 810 ± 93 ng/mL, p = 0.0368; Fig. 1E).

### 3.2 Fibroblast-specific CCN2 deletion using a tamoxifen-inducible Cre recombinase driven by the Col1α2 promoter

**Fig. 2:**
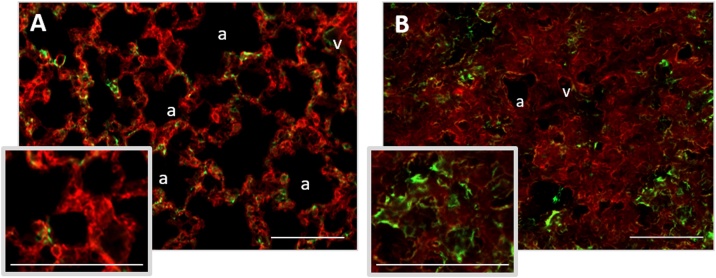
Dual fluorescence reporter mice show tamoxifen-inducible Cre recombinase activity in lung tissue and changes in lung architecture following bleomycin treatment. Representative fluorescence images of double transgenic adult Col1α2-R26TmG reporter mice containing the mT/mG reporter construct (Muzumdar et al., 2007) and Col1α2-CreERT2 gene (Li et al., 2017). Tamoxifen was administered to all mice on days 1, 3 & 5. After a 7-day interval, mice were left untreated (A), or administered a single oropharyngeal aspiration challenge of bleomycin (0.12 U/mouse; B). Lungs were isolated 14 days after bleomycin treatment. Col1α2-CreERT2 activity is indicated by an EGFP signal (green; FITC 519 nm fluorescence channel) while the tdTomato signal (red; mCherry 610 nm fluorescence channel) provides an outline of general cell morphology. Tissues were imaged using a Zeiss Axio Observer apotome microscope and Axio Cam MR R3 camera. Inserts show enlarged areas of image. Scale bars represent 100 μm.

Before using the Cre/loxP system for inducible CCN2 KO in mice, activity of the Cre recombinase in lung tissue following tamoxifen induction and bleomycin treatment was assessed, using the double fluorescent Cre reporter Col1α2-R26TmG mouse. Inducible Col1α2-CreER^T2^ activity was demonstrated by detection of an EGFP (green) signal in lung tissue from tamoxifen-treated mice (Fig. 2A&B). EGFP expression was detected in cells within bronchioles, cells lining alveoli and perivascular cells. Compared to lungs not treated with bleomcyin (Fig. 2A), bleomycin-treated lungs (Fig. 2B) displayed increased regions with tdTomato (red) signal, indicating changes to the overall lung architecture. Furthermore, regions of EGFP and tdTomato colocalisation were detected.

**Fig. 3:**
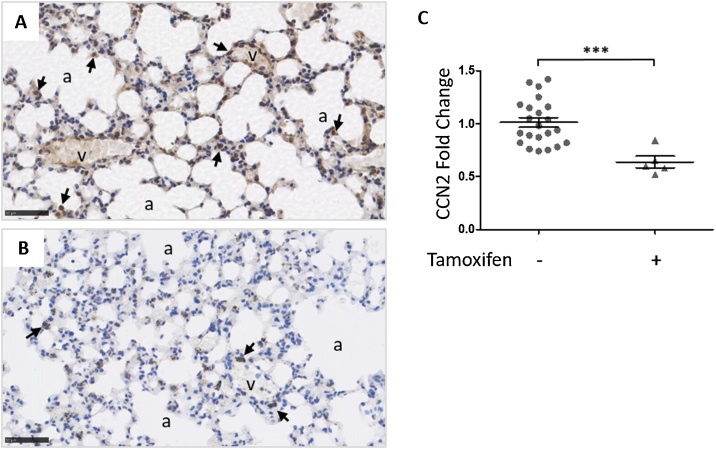
Reduced CCN2 gene expression in lung tissue following tamoxifen-induced fibroblast-specific CCN2 deletion in adult mice. Lungs were isolated from double transgenic male animals, homozygous for CCN2 flox, and positive for Col1α2-CreERT2 14 days after completion of tamoxifen dosing. Lung sections from untreated littermate control (A) and tamoxifen-induced (B) mice were stained with anti-CCN2 antibody (ab6992; black arrows). (C) RNA was extracted from whole lungs for quantitation of CCN2 mRNA expression by the ΔΔCt method of RT-qPCR analysis and normalised to 18S rRNA. Statistical significance was determined by unpaired *t*-test (*** p < 0.001). a- alveoli, v- blood vessels. Scale bars represent 50 μm.

Subsequently, to induce fibroblast-specific CCN2 deletion *in vivo*, double transgenic mice harbouring the Col1α2-CreER^T2^ construct and homozygous for CCN2 flox were treated with tamoxifen. There is a marked reduction in CCN2 expression in the structural cells within the lung following tamoxifen treatment (Fig. 3A and B). This reduction in CCN2 is mirrored by the significant reduction in CCN2 mRNA expression in the lungs of CCN2 mice (n = 5) compared to untreated control lungs (n = 22, unpaired *t*-test; p < 0.001; Fig. 3C).

### 3.3 Knockout of CCN2 in Col1α2-CreER mice abrogated bleomycin-induced pulmonary fibrosis​ and remodelling

**Fig. 4:**
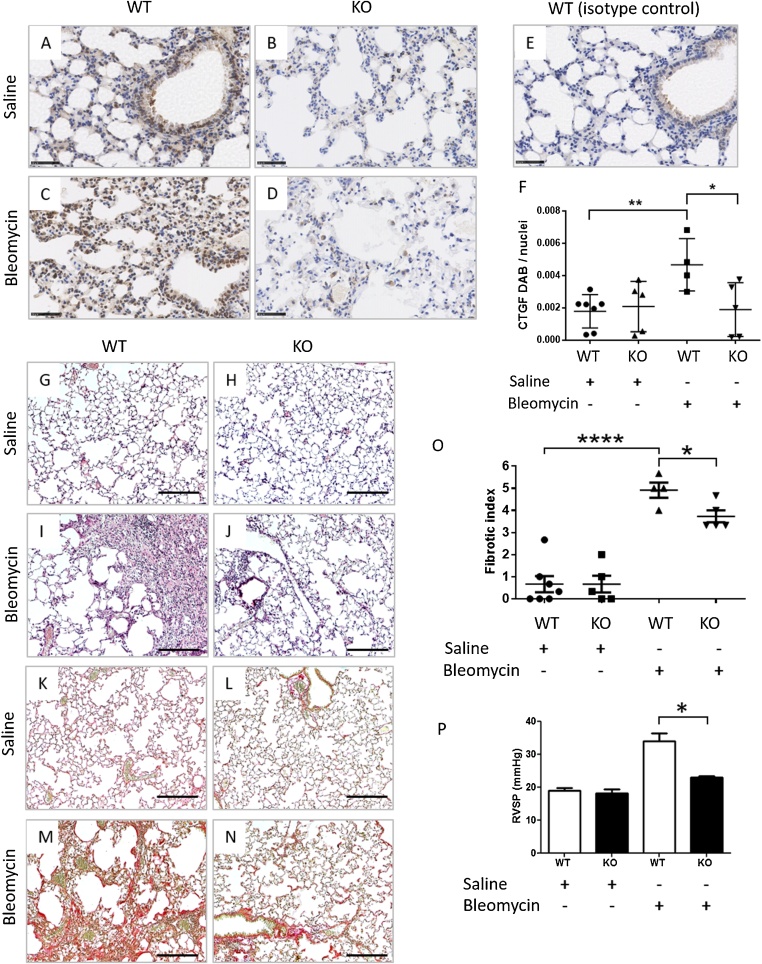
Fibroblast-specific CCN2 deletion protects mice from bleomycin-induced histological changes and pulmonary arterial hypertension (PAH). Double transgenic mice, homozygous for CCN2 flox, and positive for Col1α2-CreER, were administered tamoxifen daily for 5 days to induce CCN2 deletion (KO, n = 5 per group). Untreated littermate controls were designated as wildtype for CCN2 (WT, n = 7 for saline, n = 4 for bleomycin). After a 7 day interval, mice were treated with saline, or a single oropharyngeal instillation of bleomycin (0.12 U/mouse). After 21 days, right ventricular systolic pressure (RVSP) was measured, before lungs were harvested for CCN2 immunostaining (A–D), rabbit isotype control staining (E), hematoxylin and eosin (G–J; H&E), and picrosirius red staining (K–N). Scale bars represent 50 μm. (F) Semi-quantitation of CCN2 staining (DAB intensity / nuclei count). (O) Modified Ashcroft scoring of H&E sections to quantify extent of pulmonary fibrosis. (P) RVSP was measured for detection of PAH, sample size same as (O). Bars show mean ± S.E.M. Statistical significance was tested by unpaired *t*-test, * p < 0.05, ** p < 0.01, ****p < 0.0001. (For interpretation of the references to colour in this figure legend, the reader is referred to the web version of this article.).

To investigate the effect of fibroblast-specific CCN2 deletion on the development of lung fibrosis, we examined the extent of bleomycin-induced pulmonary remodeling, in tamoxifen-induced CCN2 KO mice. Control mice not treated with tamoxifen were designated as WT for CCN2. CCN2 staining was detected predominantly around blood vessels and bronchioles, and in the bleomycin-treated WT mice, predominantly in the expanded alveoli interstitium within the fibrotic areas. (Fig. 4A–D). Semi-quantitative analysis showed that CCN2 staining is significantly increased in the WT animals treated with bleomycin (n = 4) compared to saline (n = 7, p < 0.0001) and CTGF is significantly reduced in the bleomycin-treated KO mice (n = 5) compared to bleomycin-treated WT mice (n = 4, p < 0.05, unpaired t-tests; Fig. 4F). H&E revealed obliteration of alveoli by the presence of florid fibrosis in the lungs of bleomycin-treated WT mice and not in saline-treated WT mice (Fig. 4G–J). PicroSirius red staining showed that bleomycin-induced collagen deposition was reduced in CCN2 KO mice compared to WT mice (Fig. 4K-N). For semi-quantitative analysis, a fibrotic index was calculated for each H&E-stained lung section using the Modified Ashcroft Scale. A 20 % reduction in the fibrotic index was observed in bleomycin-treated KO lungs compared to bleomycin-treated WT lungs (unpaired *t*-test, p = 0.0277; Fig. 4O). Pulmonary pressures were significantly reduced in the bleomycin-treated KO mice compared to bleomycin-treated WT mice (p < 0.05; Fig. 4P).

**Fig. 5:**
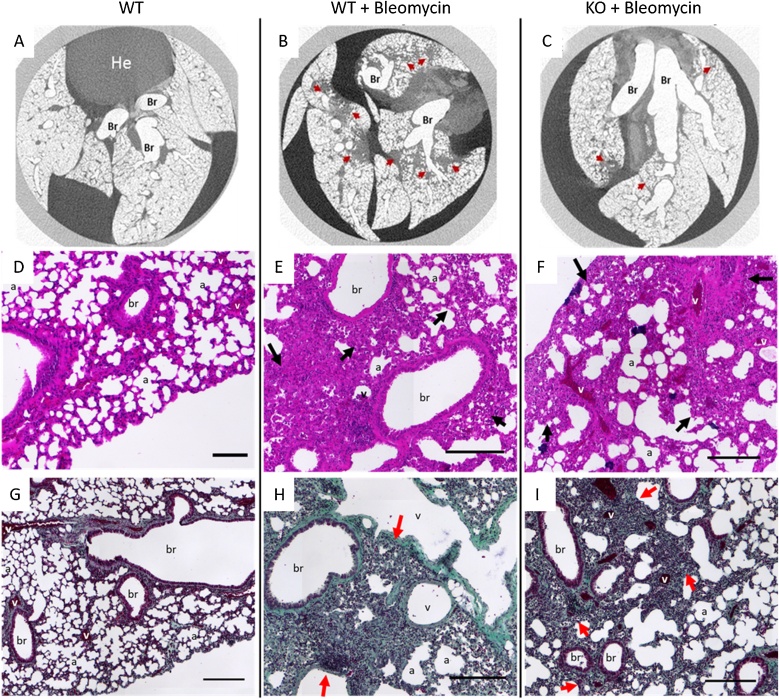
Ex vivo micro computed tomography (micro-CT) analysis shows protection against bleomycin-induced lung fibrosis in fibroblast-specific CCN2 knockout mice. Tissue analysis of double transgenic animals, homozygous for CCN2 flox, and positive for Col1α2-CreERT2 mouse lungs 14 days post-bleomycin (0.12 U/mouse) treatment. (A-C) Single plane view of micro-CT scan of inflated mouse lungs suspended in 3% potassium iodide and fixed in in 10 % neutral buffered formalin. Lungs were isolated and manually inflated for scanning, using a SkyScan 1272 micro-CT system at a resolution of 20 μm (0.25 mm aluminium filter, 0.3° rotation step). Images were reconstructed using Skyscan Nrecon software and visualised using DataViewer. Histological evaluation of lung samples using hematoxylin and eosin stain (H&E; D–F) and Goldner’s trichrome stain (G–I). Scale bars represent 200 μm. Anatomical features are identified as follows: a- alveoli, Br / br- bronchioles, v- blood vessels, He- heart, arrows (red/black) indicate regions of fibrosis. (For interpretation of the references to colour in this figure legend, the reader is referred to the web version of this article.).

Additionally, micro-CT imaging was performed to assess the extent of pulmonary fibrosis on the whole lung tissue prior to sectioning (Fig. 5). Multiple regions of established fibrosis were detected in the micro-CT scans of bleomycin-treated lungs (Fig. 5B), which corresponded to altered alveolar morphology identified in H&E stained sections (Fig. 5E) and increased collagen deposition, as shown by Goldner Trichrome staining (green; Fig. 5H). Regions of fibrosis concentrated around bronchioles. Bleomycin-induced fibrosis was reduced in CCN2 KO lungs compared to WT lungs (Fig. 5C, F&I).

**Fig. 6:**
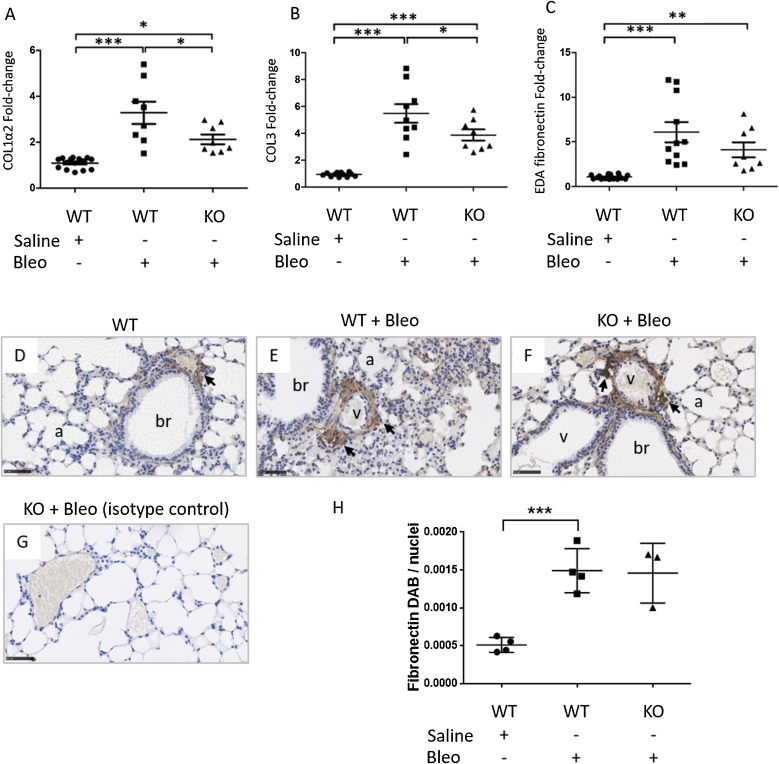
Fibroblast-specific CCN2 deletion reduces bleomycin-induced COL1α2, COL3 and EDA-fibronectin expression in mouse lungs. (A—C) RT-qPCR ΔΔCt analysis of COL1α2, COL3 and EDA fibronectin mRNA in double transgenic animals homozygous for CCN2 floxed gene and positive for fibroblast-specific Col1α2-CreERT2. Mice not treated with tamoxifen were designated as wildtype for CCN2 (WT, n = 15 for saline, n = 8 for bleomycin). Mice administered tamoxifen on days 1, 3 and 5 to induce CCN2 deletion were designated CCN2 knockout (KO, n = 8). After a 7 day interval a single oropharyngeal aspiration challenge of bleomycin (0.12 U/mouse; Bleo) was administered. Lungs were isolated 14 days post-bleomycin challenge. ΔΔCt fold-change are normalised to 18S rRNA as a reference gene. Significance was determined using one-way ANOVA followed by Tukey’s post-hoc test. (D—F) Fibronectin immunohistochemical staining of mouse lung tissue and (G) mouse IgG isotype control staining. Scale bars represent 50 μm, a- alveoli, br – bronchioles, v- blood vessels. (H) Semi-quantitation of fibronectin DAB staining normalised to haematoxilyn stained nuclei count. Unpaired *t*-test (* p < 0.05, ** p < 0.01, *** p < 0.001). WT (n = 4 per group), KO (n = 3).

To investigate the effect of CCN2 deletion on ECM production, we examined the expression of COL1α2, COL3 and EDA-fibronectin mRNA, which are precursors of ECM proteins associated with fibrotic lung phenotypes (Fig. 6A–C). Analysis of WT animals identified statistically significant upregulation of COL1α2, COL3 and EDA-fibronectin mRNA in bleomycin-treated lungs (n = 8) compared to untreated (n = 15, one-way ANOVA, and Tukey’s post hoc test, p < 0.0001). Bleomycin-induced COL1α2 and COL3 mRNA were reduced in CCN2 KO lungs (n = 8) compared to WT lungs (n = 8, p < 0.05). Fibronectin expression is significantly elevated in pulmonary tissue of WT mice following bleomycin treatment (p < 0.001) and is predominantly located around blood vessels, bronchioles and the alveolar interstitium. Localisation of fibronectin in CCN2-deleted animals is similar to that of bleomycin-treated WT animals, with perivascular and peribronchial expression (Fig. 6D–H). The changes in the pattern of fibronectin protein expression are similar to those observed by RT-qPCR.

### 3.4 Fibroblast-specific CCN2 knockout alters hypoxia/SU5416-induced tissue remodelling and increase in pulmonary pressure

**Fig. 7:**
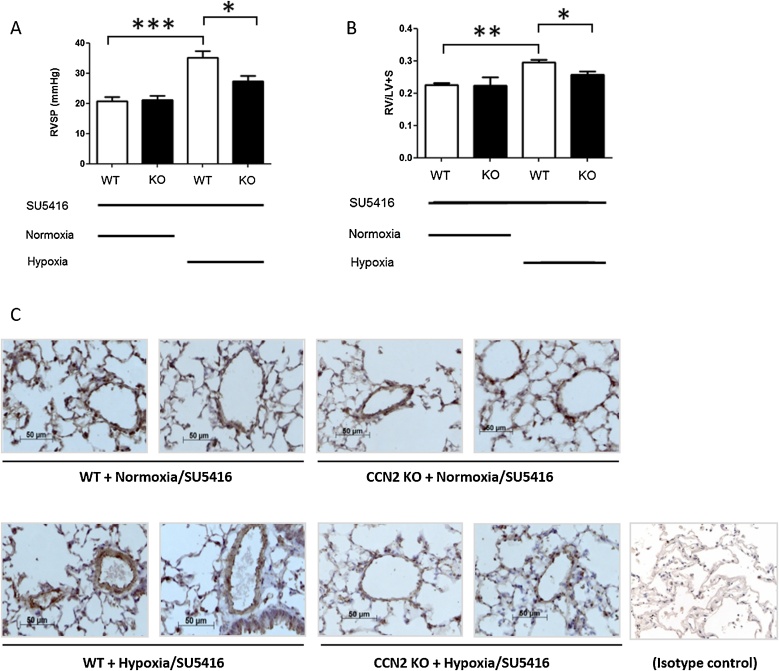
Fibroblast-specific deletion of CCN2 alters hypoxia/SU5416-induced tissue remodelling and increase in pulmonary pressure. After 21 days of hypoxia and SU5416 treatment, mice were anesthetized and pulmonary pressures measured using a catheter. (A) Right ventricular systolic pressure (RVSP). Mice not treated with tamoxifen were designated as wildtype for CCN2 (WT). Mice were treated with tamoxifen to induce CCN2 deletion (KO). Hypoxia/SU5416 induced elevated RVSP compared to normoxia/SU5416. In CCN2-null mice (KO), RVSP was significantly reduced (p < 0.05) compared to WT mice, under hypoxic/SU5416 conditions. (B) Hypoxia/SU5416 induced a significant increase (p < 0.01) in right ventricular hypertrophy (right ventricle : left ventricle plus septum ratio, RV/LV + S) in WT mice. Knocking out CCN2 significantly reduced hypoxia/SU5416-induced RV/LV + S (p < 0.05). Significance was tested by unpaired *t*-test (* p < 0.05, ** p < 0.01, *** p < 0.001; n = 6 per group). (C) Representative light microscopic images of α-smooth muscle actin (α-SMA)-stained lung sections showing a pathological pulmonary vascular phenotype with medial thickening in mice exposed to hypoxia and SU5416. Scale bars represent 50 μm. Mouse IgG isotype control-stained lung section (bottom right).

Since CCN2 KO mice showed reduced arterial pressure, indicated by reduced RVSP, in the bleomycin model, we also investigated the impact of CCN2 gene deletion in another lung disease model: a severe model of PAH, which is induced by hypoxia and Sugen/SU5416, an inhibitor of vascular endothelial growth factor (VEGF). After 21 days of hypoxia and SU5416 treatment, hemodynamic measurements and histological analysis revealed vascular changes recapitulating PAH. We sought to determine whether removal of CCN2 could protect the mice from this hypoxia/Su5416-induced PAH phenotype. In WT mice, hypoxia/SU5416 induced elevated RVSP (p < 0.001) compared to normoxia/SU5416. In CCN2 KO mice, RVSP was significantly reduced (p < 0.05) compared to WT mice, under hypoxic/SU5416 conditions (Fig. 7A). Hypoxia/SU5416 induced a significant increase (p < 0.01) in right ventricular hypertrophy (right ventricle:left ventricle plus septum ratio, RV/LV + S). Knocking out CCN2 significantly reduced hypoxia/SU5416-induced RV/LV + S (p < 0.05; Fig. 7B). Representative light microscopic images of lung sections showed a pathological pulmonary vascular phenotype with medial thickening in WT mice exposed to hypoxia/SU5416, and a reduction in vessel remodelling in CCN2 KO mice (Fig. 7C).

## 4 Discussion

In the absence of an effective anti-fibrotic treatment for SSc and other fibrotic diseases, the aims of this study were to establish whether inhibiting or removing CCN2 gene expression could provide an avenue for blocking the development of a fibrotic phenotype in the lung. We found that using siRNA to inhibit CCN2 mRNA expression in human SSc lung fibroblasts reduced constitutive expression of fibronectin, and TGF-β-induced expression of other fibrotic markers. We also found that fibroblast-specific CCN2 knockout mice showed significant resistance to bleomycin-induced pulmonary fibrosis and pulmonary hypertension.

SSc is characterised by multi-organ fibrosis with early studies providing evidence of an important role for CCN2, particularly in the development of skin fibrosis (Jimenez et al., 2010). In this study we focused on the role of CCN2 in lung fibrosis as pulmonary involvement is the leading cause of death in SSc (Nihtyanova and Denton, 2020). Therefore, we examined the expression of various fibrotic protein markers in cultured SSc lung fibroblasts, a key effector cell type in fibrosis (Garrett et al., 2017). We observed constitutive overexpression of: CCN2; fibronectin, a cofactor for TGF-β-induced myofibroblast transdifferentiation; and α-SMA, a myofibroblast marker. This is consistent with previous studies reporting dysregulated expression of these proteins in fibroblasts explanted from SSc skin (Shi-Wen et al., 2000) and SSc lungs (Hsu et al., 2011; Lindahl G.E. et al., 2013; Renzoni et al., 2004). This upregulation of CCN2 in SSc fibroblasts provided support for CCN2 as a pro-fibrotic mediator and to prove this concept, we examined whether knocking down CCN2 expression using siRNA could attenuate the fibrotic phenotype *in vitro*. We found that knocking down CCN2 reduced constitutive fibronectin expression by SSc lung fibroblasts. Cells were also treated with TGF-β as it is a potent inducer of CCN2 expression and fibroblast activation. Downregulation of CCN2 following siRNA treatment was most apparent in TGF-β-treated SSc fibroblasts. Consequently, significant reductions in fibronectin, collagen type I, α-SMA, TIMP-1 and IL-6 were observed in TGF-β-treated SSc fibroblasts following treatment with CCN2-targeting siRNA. These results may explain previous observations of concomitant upregulation of CCN2 and other fibrotic proteins such as collagen, α-SMA and IL-6 in SSc tissues (Abraham et al., 2007; Igarashi et al., 1995; Khan et al., 2012). These results provides further support that CCN2 plays an essential role in the activation of adult lung fibroblasts into α-SMA-expressing myofibroblasts, which display a contractile phenotype that contribute to the stiffness of fibrotic tissue (Kennedy et al., 2007).

As a matricellular protein, CCN2 may also contribute to tissue stiffness through its interactions with multi-ligand receptors, integrins and other ECM components (Lau, 2016). CCN2 binding to integrins can trigger Rho/Rac GTPase signalling pathways, which are involved in regulating matrix and vascular stiffness (Chaqour, 2020). Hence CCN2 has been associated with and used as a marker in conditions with high tissue stiffness such as various organ fibrosis, atherosclerosis and hypertension. The relative levels of CCN2 and other members of the CCN family of matricellular proteins are important in the regulation of tissue remodelling and fibrosis (Perbal, 2018). Indeed expression of CCN2, which is pro-fibrotic, is often inversely related to that of CCN3, which is anti-fibrotic; TGF-β treatment of dermal fibroblasts has been shown to induce CCN2 expression while downregulating CCN3 expression (Peidl et al., 2019). Furthermore, overexpression of CCN3 in fibroblasts blocks the ability of TGF-β to induce CCN2, although the molecular mechanism(s) underlying this inhibition is unknown.

Previous work showed that fibroblast-specific overexpression of CCN2 in mice resulted in a fibrotic phenotype (Sonnylal et al., 2010). Following on from this, we were interested in whether the opposite, fibroblast-specific deletion of CCN2 in mice, could provide protection against the development of fibrosis. Fibroblast-specific CCN2 deletion has been previously shown to provide resistance to mouse models of skin fibrosis induced by bleomycin (Leask and Abraham, 2006; Liu et al., 2011) or angiotensin-II (Makino et al., 2017). The data from the present study show that fibroblast-specific CCN2 deletion protects against bleomycin-induced lung fibrosis and pulmonary hypertension. CCN2 KO mouse lungs exhibited reduced ECM deposition, partial restoration of lung architecture, reduced pulmonary pressure and lower levels of COL1α1, COL3, and fibronectin gene expression than WT animals, following bleomycin challenge. Furthermore, the deletion of CCN2 from other cell types may be relevant for the treatment of other diseases. For example, myofibroblast-specific CCN2 deletion protected mice from development of a muscular dystrophy phenotype by altering collagen organisation and improving muscle regeneration (Petrosino et al., 2019). However, in a mouse model of osteoarthritis (OA), chondrocyte-specific CCN2 deletion was not found to be protective against the cartilage degeneration that associated with OA (Keenan et al., 2020). Together these data confirm that CCN2 plays a key role in promoting fibrosis and suggests that targeting CCN2 may be a viable option, at least for the treatment of human lung disease.

A potential therapeutic approach in human disease is the use of anti-CCN2 antibodies, the benefits of which have been demonstrated in pre-clinical models of fibrosis. For example, mice treated with a neutralizing anti‐CCN2 antibody, pIgY3, after bleomycin lung injury, showed reduced activity of the COL1α2 promoter, rescued pathological changes to lung architecture and reduced expression of collagen type I and α-SMA (Ponticos et al., 2009). In the study by Makino et al. (2017), the effect of pamrevlumab, (FG-3019), a human monoclonal antibody that inhibits the activity of CCN2, was comparable to that of CCN2 gene deletion in attenuating skin fibrosis in mice. Similarly, in a rat model of skeletal muscle fibrosis, blockade of CCN2 signalling by administration of pamrevlumab reduced the fibrotic phenotype including decreased α-SMA and collagen, and restored levels of the anti-fibrotic protein CCN3 (Barbe et al., 2020). In IPF, where the current therapies pirfenidone and nintedanib are unable to stop disease progression, pamrevlumab is being investigated as a potential therapy and is currently in phase 3 clinical trials (Sgalla et al., 2020). Additionally, anti-CCN2 antibodies may be applicable in other diseases such as cancer; pamrevlumab was shown to inhibit pro-tumour growth cross-talk between carcinoma cells and hepatic stellate cells (Makino et al., 2018). This antibody is also in phase 3 trials for the treatment of pancreatic cancer, and in phase 2 trials for Duschenne's muscular dystrophy (Leask, 2020). Therefore, targeting CCN2 using an anti-CCN2 antibody approach may also be a feasible option for the treatment of lung fibrosis in SSc patients.

## 5 Conclusions

The work from this study provides support for blockade of CCN2 expression or activity as a feasible therapeutic option for pulmonary fibrosis and pulmonary hypertension such as that in SSc and other diseases where CCN2 is overexpressed and plays a key role in pathogenesis.

## Funding

This work was supported by the following grants: 10.13039/501100012041Versus Arthritis (programme grant: 19472; project grants: 14376, 18627, 21810), 10.13039/501100011856MRC (programme grant: G0801052/1), The Royal Free Charity, The Rosetrees Trust, Scleroderma and Raynaud’s UK. Versus Arthritis (18672) supported Angela Tam on her PhD studentship. The Crossley Barnes Bequest supported Amy Horwell during her PhD studies.

## CRediT authorship contribution statement

**Angela Y.Y. Tam:** Conceptualization, Methodology, Formal analysis, Investigation, Writing - original draft, Writing - review & editing. **Amy L. Horwell:** Conceptualization, Methodology, Formal analysis, Investigation. **Sarah L. Trinder:** Investigation. **Korsa Khan:** Resources, Investigation. **Shiwen Xu:** Resources. **Voon Ong:** Resources, Writing - review & editing. **Christopher P. Denton:** Resources, Writing - review & editing, Funding acquisition. **Jill T. Norman:** Supervision, Writing - review & editing, Funding acquisition. **Alan M. Holmes:** Conceptualization, Writing - review & editing. **George Bou-Gharios:** Conceptualization, Funding acquisition. **David J. Abraham:** Conceptualization, Writing - review & editing, Funding acquisition.

## References

[bib0005] Abraham D. (2008). Connective tissue growth factor: growth factor, matricellular organizer, fibrotic biomarker or molecular target for anti-fibrotic therapy in SSc?. Rheumatology (Oxford).

[bib0010] Abraham D., Lupoli S., McWhirter A., Plater-Zyberk C., Piela T.H., Korn J.H., Olsen I., Black C. (1991). Expression and function of surface antigens on scleroderma fibroblasts. Arthritis Rheum..

[bib0015] Abraham D.J., Eckes B., Rajkumar V., Krieg T. (2007). New developments in fibroblast and myofibroblast biology: implications for fibrosis and scleroderma. Curr. Rheumatol. Rep..

[bib0020] Adler S.G., Schwartz S., Williams M.E., Arauz-Pacheco C., Bolton W.K., Lee T., Li D., Neff T.B., Urquilla P.R., Sewell K.L. (2010). Phase 1 study of anti-CTGF monoclonal antibody in patients with diabetes and microalbuminuria. Clin. J. Am. Soc. Nephrol..

[bib0025] Barbe M.F., Hilliard B.A., Amin M., Harris M.Y., Hobson L.J., Cruz G.E., Popoff S.N. (2020). Blocking CTGF/CCN2 reduces established skeletal muscle fibrosis in a rat model of overuse injury. FASEB J..

[bib0030] Chaqour B. (2020). Caught between a “Rho” and a hard place: are CCN1/CYR61 and CCN2/CTGF the arbiters of microvascular stiffness?. J. Cell Commun. Signal..

[bib0035] Ciuclan L., Bonneau O., Hussey M., Duggan N., Holmes A.M., Good R., Stringer R., Jones P., Morrell N.W., Jarai G., Walker C., Westwick J., Thomas M. (2011). A novel murine model of severe pulmonary arterial hypertension. Am. J. Respir. Crit. Care Med..

[bib0040] Crowe A.R., Yue W. (2019). Semi-quantitative determination of protein expression using immunohistochemistry staining and analysis: an integrated protocol. Bio Protoc..

[bib0045] Denton C.P., Khanna D. (2017). Systemic sclerosis. Lancet.

[bib0050] Denton C.P., Zheng B., Evans L.A., Shi-wen X., Ong V.H., Fisher I., Lazaridis K., Abraham D.J., Black C.M., de Crombrugghe B. (2003). Fibroblast-specific expression of a kinase-deficient type II transforming growth factor beta (TGFbeta) receptor leads to paradoxical activation of TGFbeta signaling pathways with fibrosis in transgenic mice. J. Biol. Chem..

[bib0055] Derrett-Smith E.C., Dooley A., Gilbane A.J., Trinder S.L., Khan K., Baliga R., Holmes A.M., Hobbs A.J., Abraham D., Denton C.P. (2013). Endothelial injury in a transforming growth factor beta-dependent mouse model of scleroderma induces pulmonary arterial hypertension. Arthritis Rheum..

[bib0060] Doherty H.E., Kim H.S., Hiller S., Sulik K.K., Maeda N. (2010). A mouse strain where basal connective tissue growth factor gene expression can be switched from low to high. PLoS One.

[bib0065] Dziadzio M., Usinger W., Leask A., Abraham D., Black C.M., Denton C., Stratton R. (2005). N-terminal connective tissue growth factor is a marker of the fibrotic phenotype in scleroderma. QJM.

[bib0070] Garrett S.M., Baker Frost D., Feghali-Bostwick C. (2017). The mighty fibroblast and its utility in scleroderma research. J. Scleroderma Relat. Disord..

[bib0075] Good R.B., Gilbane A.J., Trinder S.L., Denton C.P., Coghlan G., Abraham D.J., Holmes A.M. (2015). Endothelial to mesenchymal transition contributes to endothelial dysfunction in pulmonary arterial hypertension. Am. J. Pathol..

[bib0080] Hasegawa M. (2016). Biomarkers in systemic sclerosis: their potential to predict clinical courses. J. Dermatol..

[bib0085] Henrot P., Truchetet M.E., Fisher G., Taieb A., Cario M. (2019). CCN proteins as potential actionable targets in scleroderma. Exp. Dermatol..

[bib0090] Howell D.C., Goldsack N.R., Marshall R.P., McAnulty R.J., Starke R., Purdy G., Laurent G.J., Chambers R.C. (2001). Direct thrombin inhibition reduces lung collagen, accumulation, and connective tissue growth factor mRNA levels in bleomycin-induced pulmonary fibrosis. Am. J. Pathol..

[bib0095] Hubner R.H., Gitter W., El Mokhtari N.E., Mathiak M., Both M., Bolte H., Freitag-Wolf S., Bewig B. (2008). Standardized quantification of pulmonary fibrosis in histological samples. Biotechniques.

[bib0100] Igarashi A., Nashiro K., Kikuchi K., Sato S., Ihn H., Grotendorst G.R., Takehara K. (1995). Significant correlation between connective tissue growth factor gene expression and skin sclerosis in tissue sections from patients with systemic sclerosis. J. Invest. Dermatol..

[bib0105] Jimenez S.A., Castro S.V., Piera-Velazquez S. (2010). Role of growth factors in the pathogenesis of tissue fibrosis in systemic sclerosis. Curr. Rheumatol. Rev..

[bib0110] Jun J.I., Lau L.F. (2011). Taking aim at the extracellular matrix: CCN proteins as emerging therapeutic targets. Nat. Rev. Drug Discov..

[bib0115] Keenan C.M., Ramos-Mucci L., Kanakis I., Milner P.I., Leask A., Abraham D., Bou-Gharios G., Poulet B. (2020). Post-traumatic osteoarthritis development is not modified by postnatal chondrocyte deletion of Ccn2. Dis. Model. Mech..

[bib0120] Kennedy L., Liu S., Shi-Wen X., Chen Y., Eastwood M., Sabetkar M., Carter D.E., Lyons K.M., Black C.M., Abraham D.J., Leask A. (2007). CCN2 is necessary for the function of mouse embryonic fibroblasts. Exp. Cell Res..

[bib0125] Khan K., Xu S., Nihtyanova S., Derrett-Smith E., Abraham D., Denton C.P., Ong V.H. (2012). Clinical and pathological significance of interleukin 6 overexpression in systemic sclerosis. Ann. Rheum. Dis..

[bib0130] Lau L.F. (2016). Cell surface receptors for CCN proteins. J. Cell Commun. Signal..

[bib0135] Leask A. (2017). CCN2 in skin fibrosis. Methods Mol. Biol..

[bib0140] Leask A. (2020). Slow train coming: an anti-CCN2 strategy reverses a model of chronic overuse muscle fibrosis. J. Cell Commun. Signal..

[bib0145] Leask A., Abraham D.J. (2006). All in the CCN family: essential matricellular signaling modulators emerge from the bunker. J. Cell. Sci..

[bib0150] Li I.M.H., Horwell A.L., Chu G., de Crombrugghe B., Bou-Gharios G. (2017). Characterization of mesenchymal-fibroblast cells using the Col1a2 Promoter/Enhancer. Methods Mol. Biol..

[bib0155] Liu S., Taghavi R., Leask A. (2010). Connective tissue growth factor is induced in bleomycin-induced skin scleroderma. J. Cell Commun. Signal..

[bib0160] Liu S., Shi-wen X., Abraham D.J., Leask A. (2011). CCN2 is required for bleomycin-induced skin fibrosis in mice. Arthritis Rheum..

[bib0165] Makino K., Makino T., Stawski L., Lipson K.E., Leask A., Trojanowska M. (2017). Anti-connective tissue growth factor (CTGF/CCN2) monoclonal antibody attenuates skin fibrosis in mice models of systemic sclerosis. Arthritis Res. Ther..

[bib0170] Makino Y., Hikita H., Kodama T., Shigekawa M., Yamada R., Sakamori R., Eguchi H., Morii E., Yokoi H., Mukoyama M., Hiroshi S., Tatsumi T., Takehara T. (2018). CTGF mediates tumor-stroma interactions between hepatoma cells and hepatic stellate cells to accelerate HCC progression. Cancer Res..

[bib0175] Miller D.S.J., Schmierer B., Hill C.S. (2019). TGF-beta family ligands exhibit distinct signalling dynamics that are driven by receptor localisation. J. Cell. Sci..

[bib0180] Muzumdar M.D., Tasic B., Miyamichi K., Li L., Luo L. (2007). A global double-fluorescent Cre reporter mouse. Genesis.

[bib0185] Nihtyanova S.I., Denton C.P. (2020). Pathogenesis of systemic sclerosis associated interstitial lung disease. J. Scleroderma Relat. Disord..

[bib0190] Pakshir P., Noskovicova N., Lodyga M., Son D.O., Schuster R., Goodwin A., Karvonen H., Hinz B. (2020). The myofibroblast at a glance. J. Cell. Sci..

[bib0195] Peidl A., Perbal B., Leask A. (2019). Yin/Yang expression of CCN family members: transforming growth factor beta 1, via ALK5/FAK/MEK, induces CCN1 and CCN2, yet suppresses CCN3, expression in human dermal fibroblasts. PLoS One.

[bib0200] Perbal B. (2018). The concept of the CCN protein family revisited: a centralized coordination network. J. Cell Commun. Signal..

[bib0205] Petrosino J.M., Leask A., Accornero F. (2019). Genetic manipulation of CCN2/CTGF unveils cell-specific ECM-remodeling effects in injured skeletal muscle. FASEB J..

[bib0210] Ponticos M., Holmes A.M., Shi-wen X., Leoni P., Khan K., Rajkumar V.S., Hoyles R.K., Bou-Gharios G., Black C.M., Denton C.P., Abraham D.J., Leask A., Lindahl G.E. (2009). Pivotal role of connective tissue growth factor in lung fibrosis: MAPK-dependent transcriptional activation of type I collagen. Arthritis Rheum..

[bib0215] Qi W., Chen X., Poronnik P., Pollock C.A. (2008). Transforming growth factor-beta/connective tissue growth factor axis in the kidney. Int. J. Biochem. Cell Biol..

[bib0220] Ramazani Y., Knops N., Elmonem M.A., Nguyen T.Q., Arcolino F.O., van den Heuvel L., Levtchenko E., Kuypers D., Goldschmeding R. (2018). Connective tissue growth factor (CTGF) from basics to clinics. Matrix Biol..

[bib0225] Sato S., Nagaoka T., Hasegawa M., Tamatani T., Nakanishi T., Takigawa M., Takehara K. (2000). Serum levels of connective tissue growth factor are elevated in patients with systemic sclerosis: association with extent of skin sclerosis and severity of pulmonary fibrosis. J. Rheumatol..

[bib0230] Schindelin J., Arganda-Carreras I., Frise E., Kaynig V., Longair M., Pietzsch T., Preibisch S., Rueden C., Saalfeld S., Schmid B., Tinevez J.Y., White D.J., Hartenstein V., Eliceiri K., Tomancak P., Cardona A. (2012). Fiji: an open-source platform for biological-image analysis. Nat. Methods.

[bib0235] Sgalla G., Franciosa C., Simonetti J., Richeldi L. (2020). Pamrevlumab for the treatment of idiopathic pulmonary fibrosis. Expert Opin. Investig. Drugs.

[bib0240] Shi-wen X., Pennington D., Holmes A., Leask A., Bradham D., Beauchamp J.R., Fonseca C., du Bois R.M., Martin G.R., Black C.M., Abraham D.J. (2000). Autocrine overexpression of CTGF maintains fibrosis: RDA analysis of fibrosis genes in systemic sclerosis. Exp. Cell Res..

[bib0245] Sonnylal S., Shi-Wen X., Leoni P., Naff K., Van Pelt C.S., Nakamura H., Leask A., Abraham D., Bou-Gharios G., de Crombrugghe B. (2010). Selective expression of connective tissue growth factor in fibroblasts in vivo promotes systemic tissue fibrosis. Arthritis Rheum..

[bib0250] Sonnylal S., Xu S., Jones H., Tam A., Sreeram V.R., Ponticos M., Norman J., Agrawal P., Abraham D., de Crombrugghe B. (2013). Connective tissue growth factor causes EMT-like cell fate changes in vivo and in vitro. J. Cell. Sci..

[bib0255] Szabo Z., Magga J., Alakoski T., Ulvila J., Piuhola J., Vainio L., Kivirikko K.I., Vuolteenaho O., Ruskoaho H., Lipson K.E., Signore P., Kerkela R. (2014). Connective tissue growth factor inhibition attenuates left ventricular remodeling and dysfunction in pressure overload-induced heart failure. Hypertension.

[bib0260] Takagawa S., Lakos G., Mori Y., Yamamoto T., Nishioka K., Varga J. (2003). Sustained activation of fibroblast transforming growth factor-beta/Smad signaling in a murine model of scleroderma. J. Invest. Dermatol..

[bib0265] Takigawa M. (2018). An early history of CCN2/CTGF research: the road to CCN2 via hcs24, ctgf, ecogenin, and regenerin. J. Cell Commun. Signal..

[bib0270] Thoua N.M., Derrett-Smith E.C., Khan K., Dooley A., Shi-Wen X., Denton C.P. (2012). Gut fibrosis with altered colonic contractility in a mouse model of scleroderma. Rheumatology (Oxford).

[bib0275] van den Hoogen F., Khanna D., Fransen J., Johnson S.R., Baron M., Tyndall A., Matucci-Cerinic M., Naden R.P., Medsger T.A., Carreira P.E., Riemekasten G., Clements P.J., Denton C.P., Distler O., Allanore Y., Furst D.E., Gabrielli A., Mayes M.D., van Laar J.M., Seibold J.R., Czirjak L., Steen V.D., Inanc M., Kowal-Bielecka O., Muller-Ladner U., Valentini G., Veale D.J., Vonk M.C., Walker U.A., Chung L., Collier D.H., Ellen Csuka M., Fessler B.J., Guiducci S., Herrick A., Hsu V.M., Jimenez S., Kahaleh B., Merkel P.A., Sierakowski S., Silver R.M., Simms R.W., Varga J., Pope J.E. (2013). 2013 classification criteria for systemic sclerosis: an American college of rheumatology/European league against rheumatism collaborative initiative. Ann. Rheum. Dis..

[bib0280] Walters D.M., Kleeberger S.R. (2008). Mouse models of bleomycin-induced pulmonary fibrosis. Curr. Protoc. Pharmacol..

[bib0285] Wang Q., Usinger W., Nichols B., Gray J., Xu L., Seeley T.W., Brenner M., Guo G., Zhang W., Oliver N., Lin A., Yeowell D. (2011). Cooperative interaction of CTGF and TGF-beta in animal models of fibrotic disease. Fibrogenesis Tissue Repair.

[bib0290] Wang X., Cui H., Wu S. (2019). CTGF: a potential therapeutic target for Bronchopulmonary dysplasia. Eur. J. Pharmacol..

[bib0295] Wongkarnjana A., Scallan C., Kolb M.R.J. (2020). Progressive fibrosing interstitial lung disease: treatable traits and therapeutic strategies. Curr. Opin. Pulm. Med..

[bib0300] Yin Q., Liu H. (2019). Connective tissue growth factor and renal fibrosis. Adv. Exp. Med. Biol..

